# Ultra-long metal nanowire arrays on solid substrate with strong bonding

**DOI:** 10.1186/1556-276X-6-525

**Published:** 2011-09-09

**Authors:** Ju Xu, Lan Chen, Alan Mathewson, Kafil M Razeeb

**Affiliations:** 1 Tyndall National Institute, University College Cork, Cork, Lee Maltings, Dyke Parade, Cork, Ireland; 2 Department of Chemistry, University College Cork, Cork, Ireland

**Keywords:** nanowire arrays, anodic alumina membranes, electrodeposition, nano-interconnect

## Abstract

Ultra-long metal nanowire arrays with large circular area up to 25 mm in diameter were obtained by direct electrodeposition on metalized Si and glass substrates via a template-based method. Nanowires with uniform length up to 30 μm were obtained. Combining this deposition process with lithography technology, micrometre-sized patterned metal nanowire array pads were successfully fabricated on a glass substrate. Good adhesion between the patterned nanowire array pads and the substrate was confirmed using scanning acoustic microscopy characterization. A pull-off tensile test showed strong bonding between the nanowires and the substrate. Conducting atomic force microscopy (C-AFM) measurements showed that approximately 95% of the nanowires were electrically connected with the substrate, demonstrating its viability to use as high-density interconnect.

## Introduction

Recently, template-based methods have been successfully adopted for fabrication of metal, semiconductor and polymer nanowire arrays by electrodeposition. These methods have used heavy ion-irradiated polycarbonate (PC) film or patterned porous anodic alumina membranes (AAM) as the template [1]. AAM has the advantages of high-density, highly ordered pore channels and adjustable pore size compared to the porous PC film. The nanowire arrays obtained can be used for interconnects [2], ultrahigh density capacitors [3], batteries [4], memories [5], biosensors [6-8], etc. For all these applications, length and aspect ratio of the nanowires are the most important parameters to consider. Increasing the length of nanowires will not only enhance the surface area dramatically but also increase the amount of nano-devices grown in one single nanowire [5]. High-aspect ratio metal nanowire arrays with length above 10 μm are usually obtained by using a sputtered metal seed layer on the back of AAM template with follow-on electrodeposition [9]. However, the main disadvantage when using these metal substrates is the lack of possibility for readily integrating the nanostructure array with the current microsystem technology. Also, the metal thin film deposited on the back of the AAM is thin and soft and is not a strong physical support [10]. This is a hindrance to achieving uniform length nanowire arrays with large area coverage because mechanical polishing is required to level the unevenness of the deposited nanowire tops [11]. To address these difficulties, there have been many studies where AAMs were fabricated by anodization of evaporated or sputtered aluminium films on solid substrates, such as Si or glass [12,13]. In order to achieve long nanowires, a thick AAM is required, and this requires the use of a thick Al layer and a long anodization time [14]. The deposition of a thick Al film (above 10 μm) on a solid substrate is neither easy nor cost-effective, especially when considering the quality of the evaporated Al [15] material. However, by using the electron-beam evaporation, an Al film up to 50 μm thick [12] and a length of about 9 μm nanowire has recently been demonstrated [13], most of the publications about nanowire arrays on Si substrates show values below 3 μm [1,9,13,16]. This is due to the high cost of evaporation and thermal-stress which causes detachment during the anodization of the thick Al film. A further disadvantage of using this AAM-Si template created from an evaporated Al film is that the pores of the AAM tend not to be open at the bottom of the channels due to the insulator barrier layer between AAM and Si substrate, this results in a further step to remove the barrier layer, weak bonding between AAM and Si or even the detachment of the AAM from the Si surface have been observed if the barrier were not properly removed [17]. It would also be very difficult to achieve large area uniform nanowire arrays on a solid substrate using the AAM template prepared by the above techniques, due to the difficulty in achieving uniform Al thickness over a large area, and of maintaining appropriate homogeneity of the template's pore channels [18].

Recently, Taberna [19] attached a commercial AAM to a smooth copper foil as an electrode without using any conductive glue between AAM template and the copper foil. In this work, another thicker copper foil was put on the top of AAM and worked as an anode with a cellulose paper separator between the AAM and anode copper foil. Copper nanowire arrays, directly attached to the smooth copper foil were achieved. Theoretically, this method can also achieve ultra-long nanowires on other solid substrates, but this setup requires a flat anode which is not process friendly when mesh type anodes are required. Furthermore, it is not easy to get homogeneous nanowire arrays with a large area due to the pore blocking effect of the cellulose paper, specifically when the template's pore diameters are comparable to the width of the cellulose fibres. Nano-pillar gold arrays on a gold-coated glass substrate have also been achieved using a similar procedure [19]. However, the length of these nano-pillars was less than 1 μm. So far, no nanowire arrays with length above 9 μm on Si or glass substrate have been achieved by this template method. In this paper, we report an effective procedure using a new setup for the fabrication of ultra-long metal nanowire arrays directly onto Si and glass substrates. Both copper and silver nanowires were fabricated using this setup. The length of these nanowire arrays can be up to 30 μm with length uniformity in an area covering up to 25 mm in diameter. This technique when combined with lithography technology enabled micrometre-size-patterned nanowire array pads on a glass substrate to be fabricated. The strong bonding between nanowires and the bottom substrate was demonstrated by a tensile stress test, performed on the large area nanowires and the subsequent scanning acoustic microscopy (SAM) analysis on patterned nanowires. Furthermore, conductive atomic force microscopy analysis on the nanowire-AAM composite was performed to show the uniform conducting properties of the composite and to prove that the each wire was electrically connected to the substrate.

## Experimental details

### Electrodeposition

Electrodeposition of copper nanowires was carried out at room temperature at a constant current with a density of 1.0 mA/cm^2^. Electrodeposition usually takes about 5 to 12 h from an electrolytic bath where a typical copper bath consists of 200 g l^-1 ^of CuSO_4_.5H_2_O and 20 g l^-1 ^of H_2_SO_4 _and a silver bath consists of 16 g l^-1 ^of Ag_2_SO_4_, and 224 g l^-1 ^of potassium thiocyanate. A two electrode cell was used for deposition and copper or Ag foil was used as counter electrode. The deposition was performed at a stirring speed of 500 rpm at room temperature. The commercial AAM used in this paper are Anodisc™ membrane filter with a 200-nm nominal pore diameter from Whatman plc (Maidstone, Kent, UK). To obtain nanowire arrays on substrate, the template was removed in 6.0 M KOH, washed with plenty of de-ionised water and dried in air.

### Pull-off adhesion tensile test

The adhesion testing was carried out using an Elcometer 110 P.A.T.T.I. pneumatic tester according to the ASTM D4541-95e1 standard. A Ag nanowire-AAM on Si sample was selected to avoid the effect of oxidation of the copper nanowire tips. The sample size was 11 mm in diameter. The diameter of the test stud was 8.16 mm which is related to 0.52-cm^2 ^test area. To run the adhesion test, the stud was attached to the Ag nanowire-AAM surface with Araldite epoxy adhesive as shown in Figure S1a (see Additional file 1). The Si wafer was attached on a flat metal base plate as shown in Figure S1b (see Additional file 1). For execution of a pull-off test, the stud of the test equipment has to be firmly attached to the nanowire-AAM surface. To ensure this, the surfaces of the stud and the nanowire-AAM composite were polished using a coarse sand paper and subsequently cleaned in acetone before applying the adhesive epoxy. After the sample was mounted, the specimen under test was pulled perpendicular to the wafer substrate until some part of the structure under ruptures.

### SAM characterization

SAM characterization was carried out by SONIX HSl000 with software IC5.98d and transducers with nominal frequencies of 75 MHz. The transducer tip and sample were immersed in distilled water at room temperature. The test was performed with the Si substrate side up, in order to avoid signal scattering due to the rough surfaces of the nanowires. Signals from the sample were transferred to a personal computer for advanced numerical data analysis.

### Conductive AFM characterization

Conductive AFM characterization was performed using a Dimension D3100 scanning probe microscope. The tip used in this study was electrically conductivity probes made from 0.01 to 0.025 Ωcm antimony (n)-doped Si. The nominal tip radius is 20 nm and the front and backside of the tip was coated with 20 nm Pt/Ir on top of 3 nm Cr. (see Figure S2 in Additional file 1) The scanning parameters (scan rate = 1 Hz; integral gain = 2; proportional gain = 3 to 4; deflection set point = 1 V; DC sample bias = 1 to 2 V) were set to these values for all experiments.

## Results and discussion

**Figure 1:**
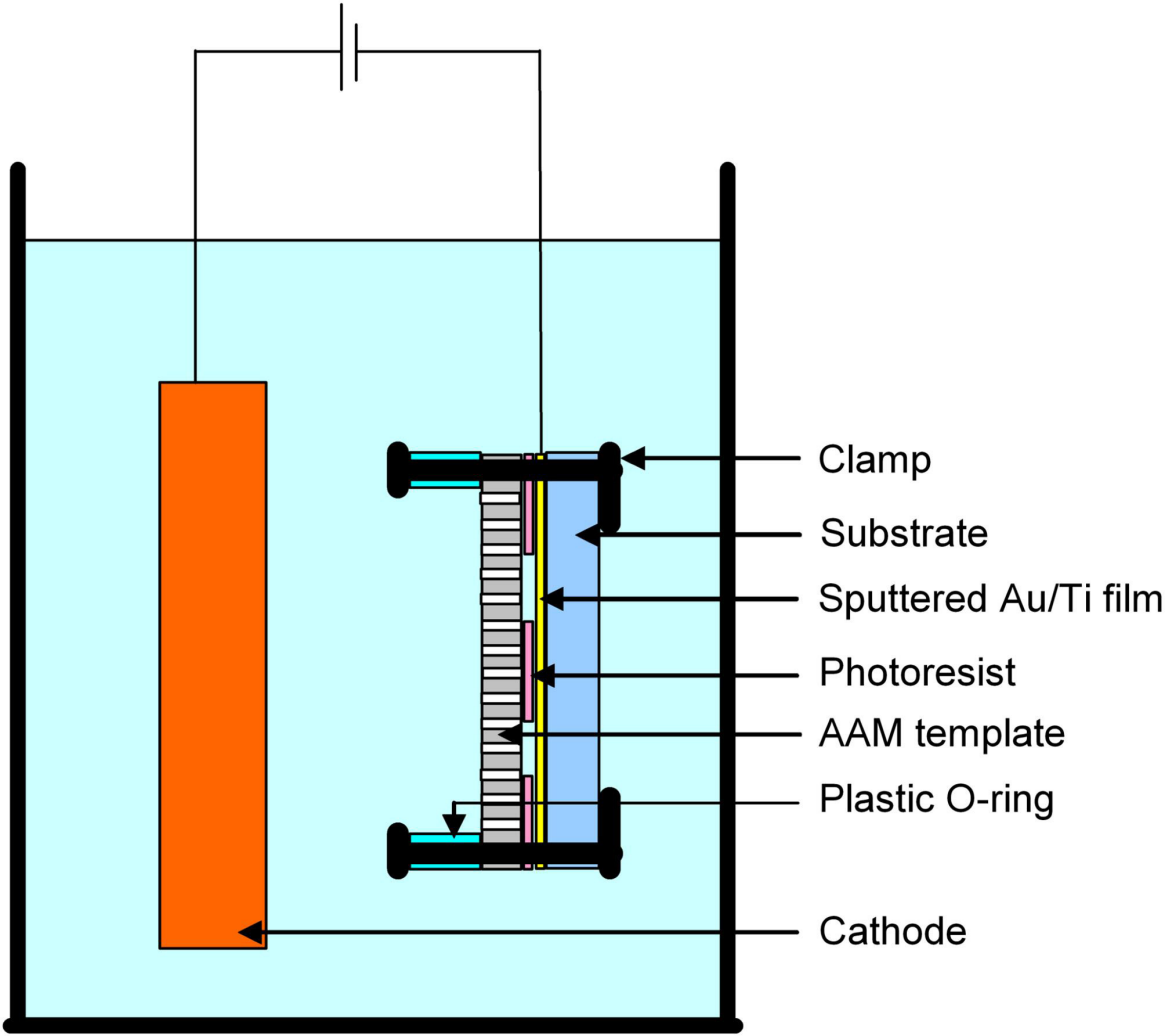
**Setup for electrodeposition**.

**Figure 2:**
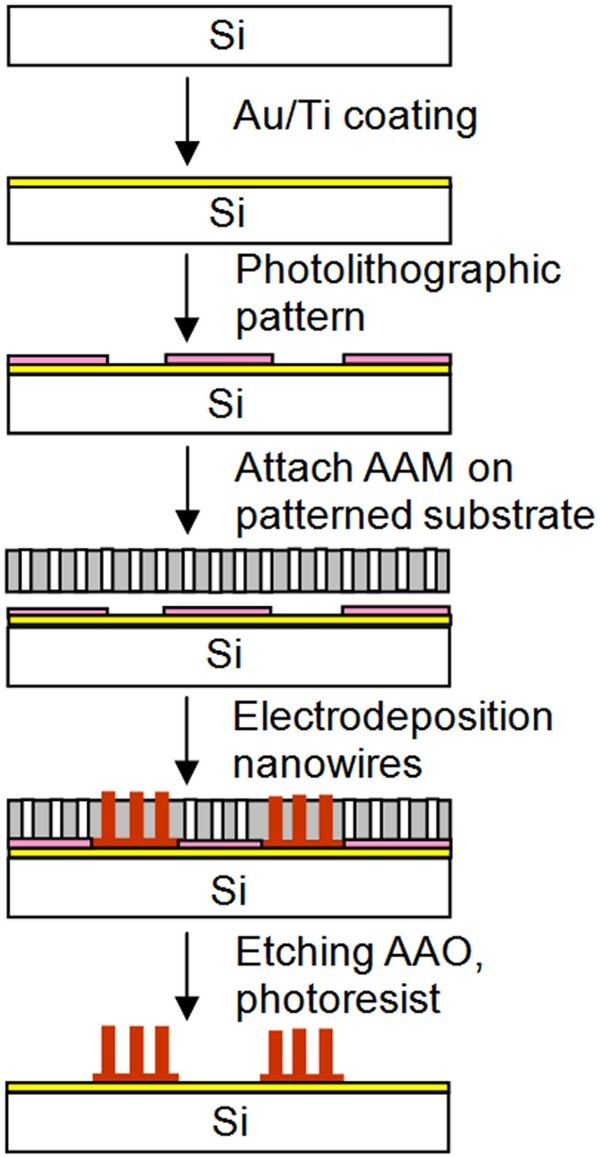
**Schematic for fabrication process of metal nanowire patterns on substrate**.

Fabrication of metal nanowire arrays on a solid substrate were performed by cathodic electrodeposition from an electrolytic bath inside a commercial AAM [20]. The AAM was directly attached to a Au/Ti-coated solid substrate (either Si or glass) using a specially designed sample holder as shown in Figure 1. A plastic O-ring was mounted on the edge of the AAM template to distribute the pressure evenly and to avoid the destruction of the AAM template due to its brittle nature. For the deposition of patterned nanowire arrays on a solid substrate, the substrate was treated using the procedures shown in Figure 2. Initially, a thin conductive layer of Au/Ti (300:20 nm) was sputtered on the Si substrate followed by spin-coating of a thin layer (about 1.2 μm) of photoresist. After exposure to UV light under a photolithographic mask, the substrate was soaked in developer solution. The patterned underlying Au layer was exposed and used as a cathode. In order to achieve non-patterned nanowires on a large surface area, lithography process is not required; however the thin conductive layer of Au/Ti is necessary as a seed layer for the growth of nanowires. What the most important in this process is to ensure that the applied pressure is even across the entire sample or substrate because this will ensure even growth of nanostructures.

**Figure 3:**
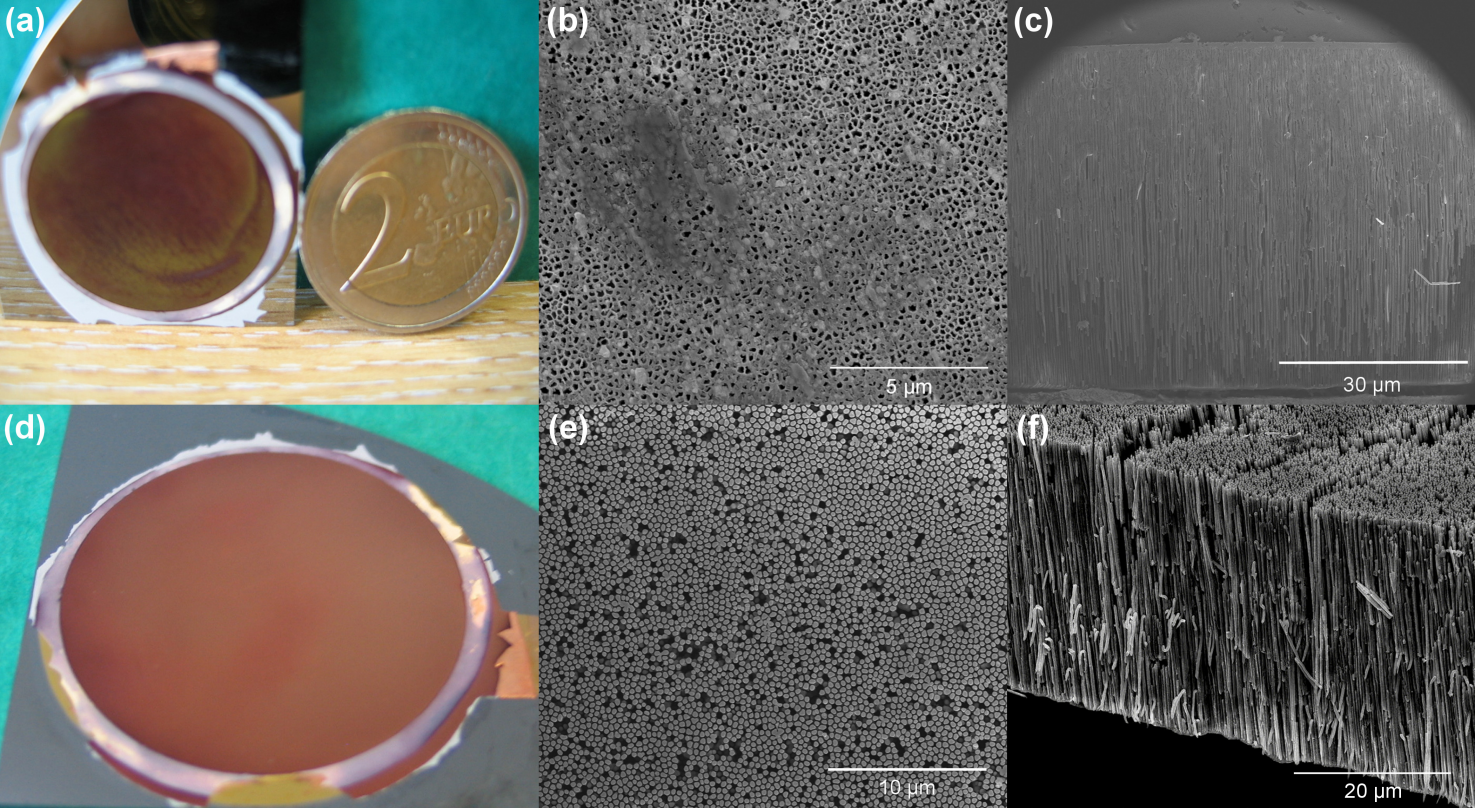
**Images of Cu nanowire arrays on Si substrate**. **(a) **Optical image of non polished sample; **(b) **top view SEM image of non polished sample showing approximately 30% pore filling; **(c) **cross sectional view SEM image of non polished sample showing the length of nanowires is about 40 to 50 μm; **(d) **optical image of polished sample shows mirror surface with a large circular area in diameter of 25 mm; **(e) **top view SEM image of polished sample with 20-μm wires down from the top surface, showing 98% pore filling; **(f) **cross sectional SEM image of polished sample showing the nanowire arrays with uniform length of approximately 30 μm after the AAM was dissolved.

Figure 3 shows the Cu nanowire arrays within the AAM template on a Si substrate before and after mechanical polishing. Nanowires within the AAM over a large circular area (25 mm in diameter) were achieved as shown in Figure 3a. The overgrowth layer was manually removed and the scanning electron microscopy (SEM) image of Figure 3b revealed that about 30% pores were fully filled according to the calculation of ratio of pores filled with nanowires to all the template pores on the top surface. This low filling ratio, which was initially observed from the top of the template, was due to the overgrowth of selected wires. The commercial template used in this study has branched pore structures [21] (on the filtration side) and therefore a few wires may reach the top of the template first, grow laterally and thereby block the unfilled pores. This will eventually stop the solution to transport into the unfilled pores and prohibit the nanowire growth inside them [11]. The diameter of the nanowires was about 209 nm which is related to the pore diameters of the AAM template [22] used. Smaller diameters of nanowires should be achievable if smaller pore sizes of template are used. The cross section image in Figure 3c shows that the length of these nanowires is about 40 to approximately 50 μm. This is associated to an aspect ratio of 200 to approximately 250 based on the wire diameter. A thin layer of metal film of approximately 2 μm thickness was found between the AAM and substrate due to the physical gap between them. The adhesion between nanowires and Si substrate is strong enough to sustain the force of mechanical polishing using a Logitech precision lapping machine. Figure 3d shows a plan view of a Cu nanowire array within an originally 50-μm-thick template after mechanically polishing down to 20 μm from the top surface. The mirror-polished large area was achieved, suggesting uniform length of nanowires were obtained. The high-resolution SEM image in Figure 3e shows that nearly 98% pore filling ratio on the top surface of template was achieved at this location in the template. It is worth noting that the filling ratio is highly dependent on the depth of surface polishing due to the unevenness of nanowire growth along the template channels from the bottom (nearly 100% pores were filled on the bottom of channels according to Figure 3c) to top. The cross sectional view of a polished sample in Figure 3f shows that nanowires with a uniform length of approximately 30 μm were achieved.

**Figure 4:**
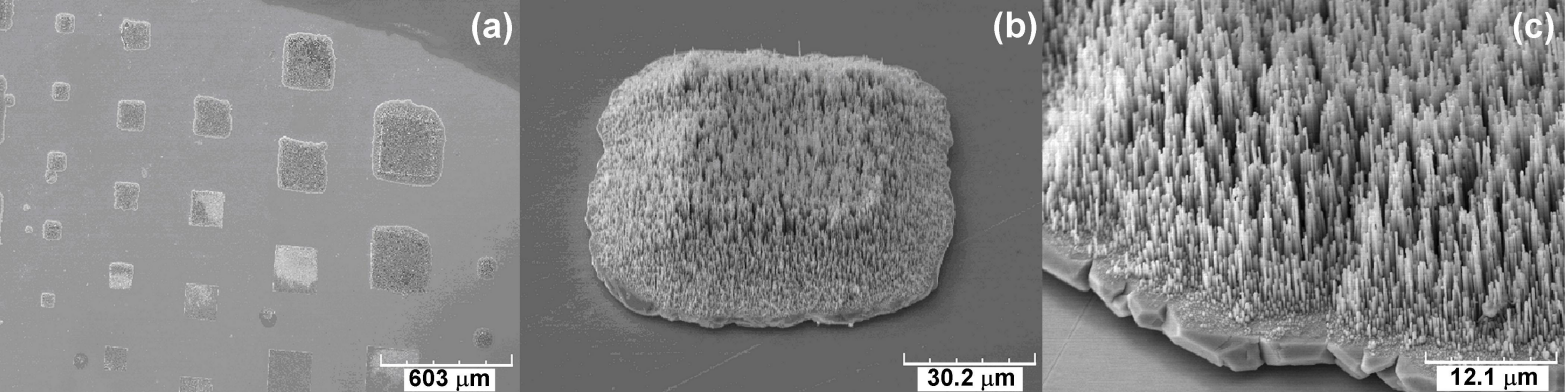
**Silver nanowire array patterns**. **(a) **Silver nanowire array patterns on glass substrate with different feature sizes; **(b) **one nanowire array pattern with size of about 60 × 60 μm; **(c) **high magnification of image (b) (45° tilt view).

The ultra-long and uniform length of these nanowires are very useful for some applications, such as *Z*-axis interconnects, because uniform lengths will allow for large surface area contact in an electronic device, leading to maximum electrical and thermal device performance. Also, this long wire length will provide enough gaps between top and bottom substrates for filling with underfill [23] for mechanical stability [24]. For further the usefulness of nanowire arrays as a *Z*-axis interconnect, the wires need to be bonded on the pre-patterned pads of the wafer/devices. Recently, micrometre-sized Ni pillars (low aspect ratio) were fabricated on top of the pads using lithography processes [25], which is time consuming and expensive to ultra-long nanowires. Alternative to this, we are proposing direct fabrication of nanowires on top of the pads based on this template attachment approach. Micrometre-sized nanowire patterns (both Cu and Ag) were fabricated on glass substrates, using the procedure shown in Figure 2. Electrodeposition had selectively taken place on the exposed metal area (which act as pads), initially defined by the lithography, due to the insulation of photoresist. After dissolving of AAM in KOH solution and stripping off the photoresist, patterned nanowire array pads on the Au/Ti-coated glass substrate were achieved. Figure 4a shows a representative image of the Ag nanowire patterns with different pad sizes. Homogeneous deposition on pad sizes from 200 × 200 to 50 × 50 μm^2 ^was achieved, while smaller features are also feasible. There is a reduction of the length of nanowires around the edge of the pads (Figure 4b), which showed similar results as the low aspect ratio nanowire array pads made by other template-based methods [9]. The specific reason for this phenomenon is unknown. Figure 4c shows a higher magnification image of a single pad where aligned nanowires and the intermediate Ag layer are visible.

**Figure 5:**
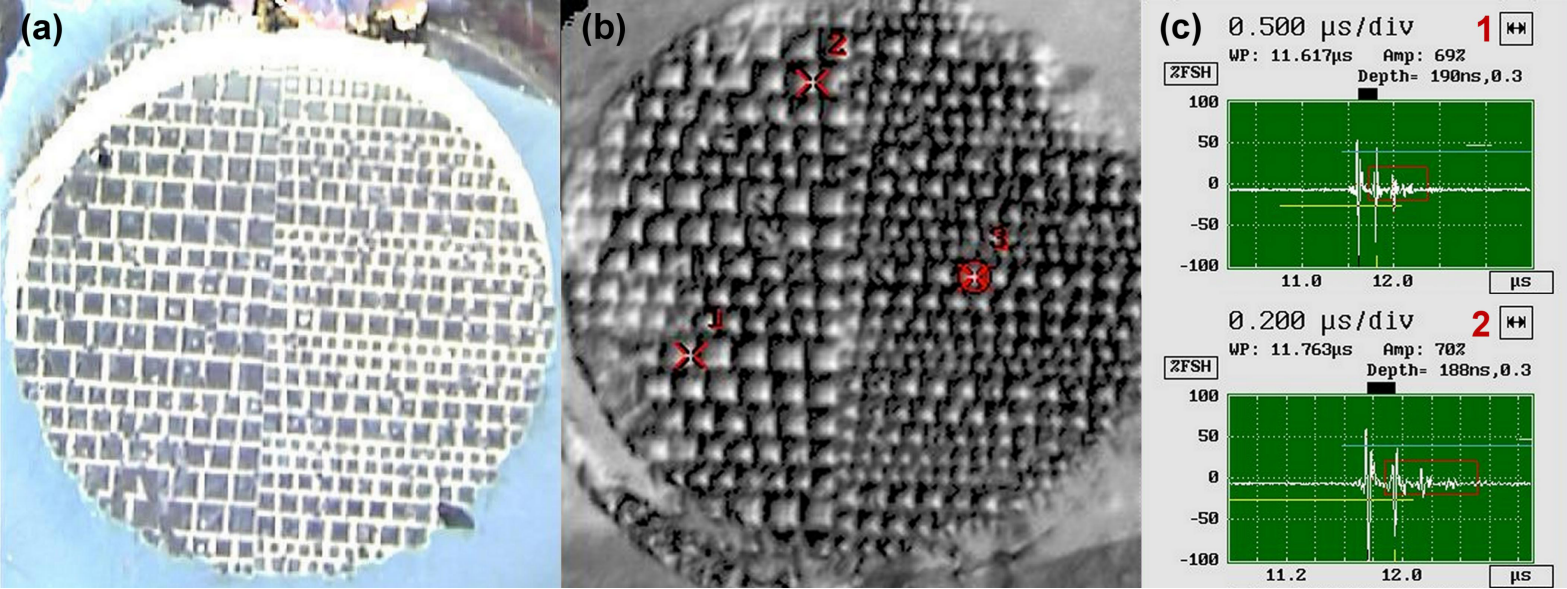
**Patterned Ag nanowire arrays on glass substrate**. **(a) **Optical image; **(b) **SAM C-scan mode image; **(c) **typical two oscilloscope traces obtained with glass substrate facing up.

The bonding integrity between nanowire/interlayer film and the Si wafer was characterised by a tensile tester. The adhesion strength between them is 38.4 MPa (3.84 kN/cm^2^) which is regarded as strong bonding [26]. Although it is not recommended that the value reported here be compared directly with those obtained by different testing methods, this interface is still much stronger than the strongest bonding of CNT arrays on Si substrate, which showed a strength of 30 to 110 N/cm^2 ^by a compression shear test [27,28]. Also, the sample was fractured at the interface of Au/Ti thin film and Si wafer (see Figure S1b in Additional file 1), suggesting comparatively stronger bonding between the nanowires and Au/Ti film than that of between Au/Ti film and Si wafer. This strong bonding between nanowires and the substrate is another big advantage for the *Z*-axis interconnects application. The bonding between Ag nanowire pads and the glass substrate were further characterised by SAM technique using a SONIX HSl000 (SONIX, Inc., Springfield, VA, USA). SAM is a non-destructive technique used extensively throughout the microelectronics industry to inspect a bonded interface for the visualisation of delamination and cracking. Figure 5a shows the optical image of Ag nanowire pads (dark area) on glass substrate, the white colour shows the empty AAM without nanowires. The related SAM image in the C-scan mode for the same sample of patterned nanowire array pads on glass substrate is shown in Figure 5b. Almost all of the nanowire pads are clearly distinguishable and appear greyish in colour, whereas the empty AAM area without Ag nanowires shows black colour, which is the typical delaminating image. Further to this, two typical oscilloscope traces are shown in Figure 5c. The traces show that about 69% and 70% waves were captured, suggesting a good adhesion between the glass substrate and all of the nanowire pads.

**Figure 6:**
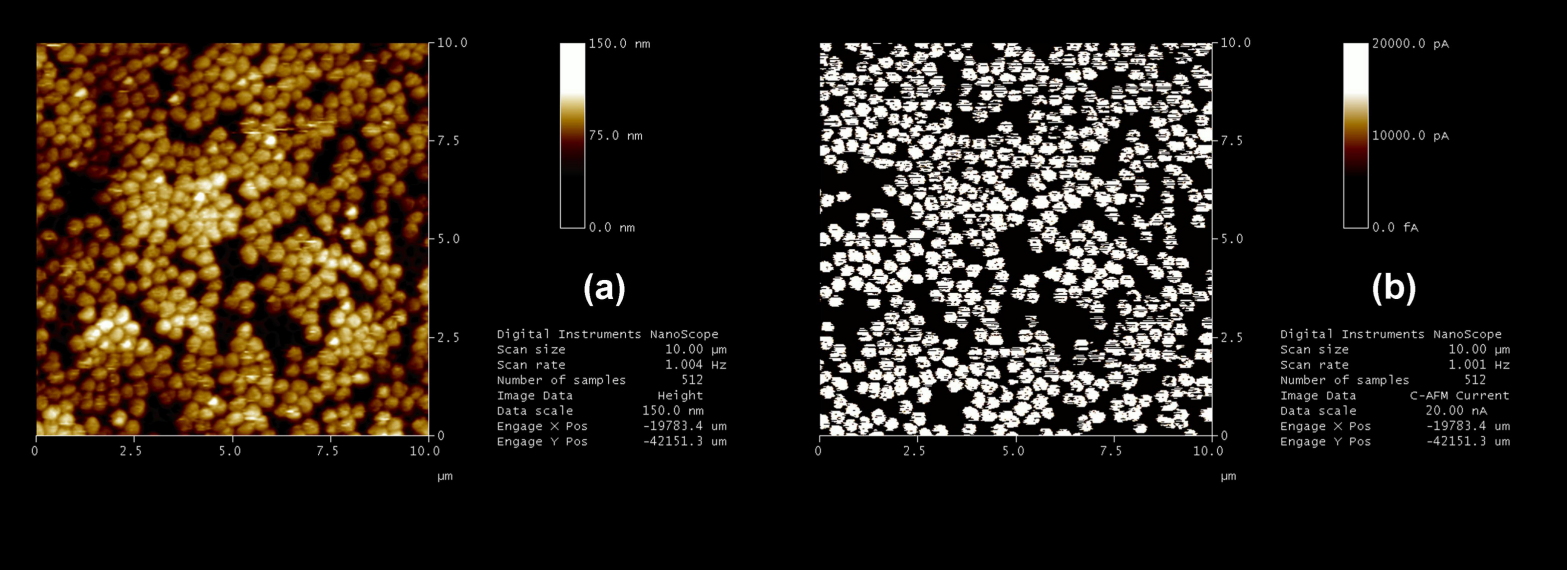
**AFM analysis for 10 × 10-μm^2 ^areas**. **(a) **Topographic images; **(b) **conductive AFM image shows nearly 95% copper nanowires are electrically conductive.

Uniform length with firm electrical connection of these nanowires on a large surface area is another important factor to be considered for applications such as *Z*-axis interconnects. Thus, the electrical connection between the mechanically polished Cu nanowire arrays and the bottom Si substrate (for the sample in Figure 3d) was further characterised using a Dimension D3100 scanning probe microscope (Veeco, Plainview, NY, USA) in conductive atomic force microscopy mode (C-AFM). The topographic and the corresponding conductive scanning images (current image) of these nanowires are shown in Figure 6. Figure 6a, b shows the topographic image for 10 × 10-μm^2 ^scan area and the corresponding current map image. Nearly 95% of the nanowires were conductive, suggesting good electrical connection between the tip of the nanowires and the bottom substrate. Different areas of the sample, from middle to the edge were scanned and a similar percentage of conductive nanowires were obtained, suggesting a large area of uniformity among these nanowires.

## Conclusions

A useful method has been developed to fabricate ultra-long metal nanowire arrays, from micrometre-size pattern to large area (of 25 mm diameter), on a solid substrate by using a ready-made AAM as template. By mechanically polishing the surface after nanowire fabrication, nanowire arrays with uniform length up to 30 μm were achieved. The nanowires and substrate were bonded via intermediate metal film layer, which showed a very good adhesion. Conducting AFM analysis showed that nearly 95% of the nanowires had good electrical connection with the substrate. While only metal nanowires fabricated on Si and glass substrate are shown in this letter, the approach described here can be adjusted to deposit any conductive materials, oxides, conductive polymers and semiconductors, on any solid substrate as well as flexible polymer substrate. In addition, by slightly changing the fabrication procedures, individually addressable nanowire pads/electrodes with micrometre size can also be fabricated (see Figure S3 in Additional file 1). It is also possible to achieve longer (higher aspect ratio) and larger area coverage of these nanowire arrays with uniform length for nearly 100% pore filling, if in-house fabricated AAM with homogenous pore distribution and larger size template is used.

## Competing interests

The authors declare that they have no competing interests.

## Authors' contributions

JX carried out the nanowire array experiment and prepared the manuscript. LC provided ideas to sample holder design for nanowire array fabrication and participated in drafting the manuscript. AM provided the ideas to use nanowire arrays on interconnect. KMR conceived the study, participated in experimental design and coordination and helped to draft the manuscript. All authors read and approved the final manuscript.

## Supplementary Material

Additional file 1 **Electronic Supplementary Material**. http://www.nanoscalereslett.com/imedia/2066074704584386/supp1.doc. Figure S1, pull-off adhesion tensile test of silver nanowire arrays within AAM template fabricated on Au-coated Si substrate; Figure S2, conductive atomic force microscopy (C-AFM) characterization; and Figure S3, process of fabricating individually addressable nanowire patterns on Si substrate. Click here for file
