# Supplementary material for: Ultra-long metal nanowire arrays on solid substrate with strong bonding
Source: Nanoscale Res Lett. 2011 Sep 9;6(1):525. doi: 10.1186/1556-276X-6-525 (PMC3212064; doi:10.1186/1556-276X-6-525)

Electronic Supplementary Material

Ultra long Metal nanowire Arrays on Solid Substrate with Strong Bonding

| Ju Xu^1^, Lan Chen^2^, Alan Mathewson^1^ and Kafil M Razeeb^1*^  ^1^ Tyndall National Institute, Lee Maltings, University College Cork, Cork, Ireland  ^2^ Department of Chemistry, University College Cork, Cork, Ireland  *Address correspondence to [kafil.mahmood@tyndall.ie](mailto:kafil.mahmood@tyndall.ie)  Supporting information to DOI |
| --- |

1. Pull-off adhesion tensile test of silver nanowire arrays within AAM template fabricated on Au coated Si substrate a) Schematic of test set up b) Image of sample detached from Si substrate, showing that the fracture was occurred at the interface of Ti and Si substrate.


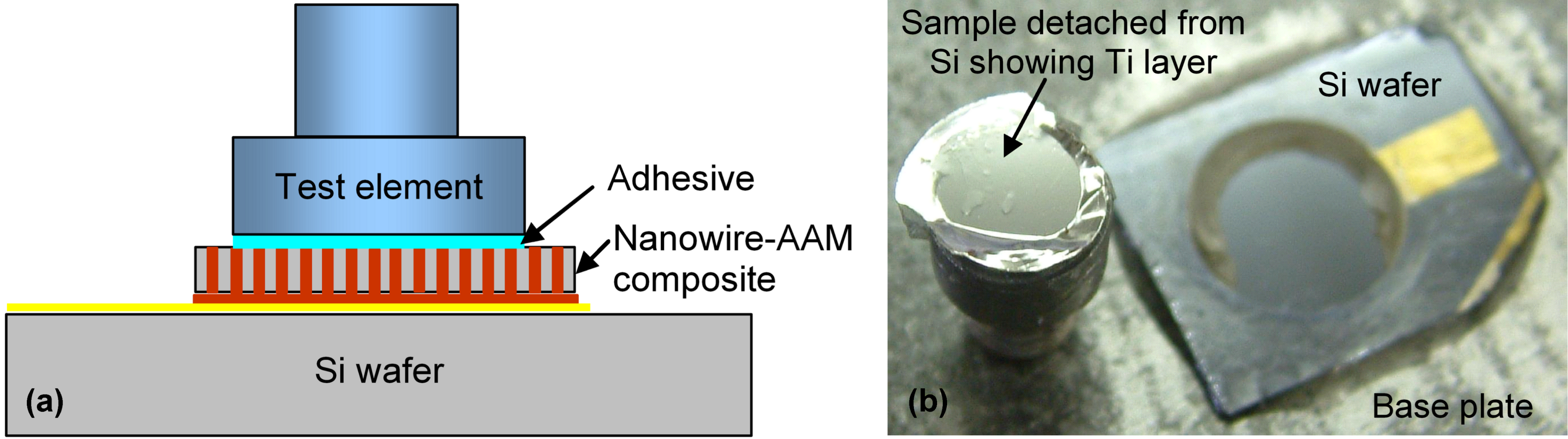


1. Schematic showing conductive atomic force microscopy (C-AFM) characterization.


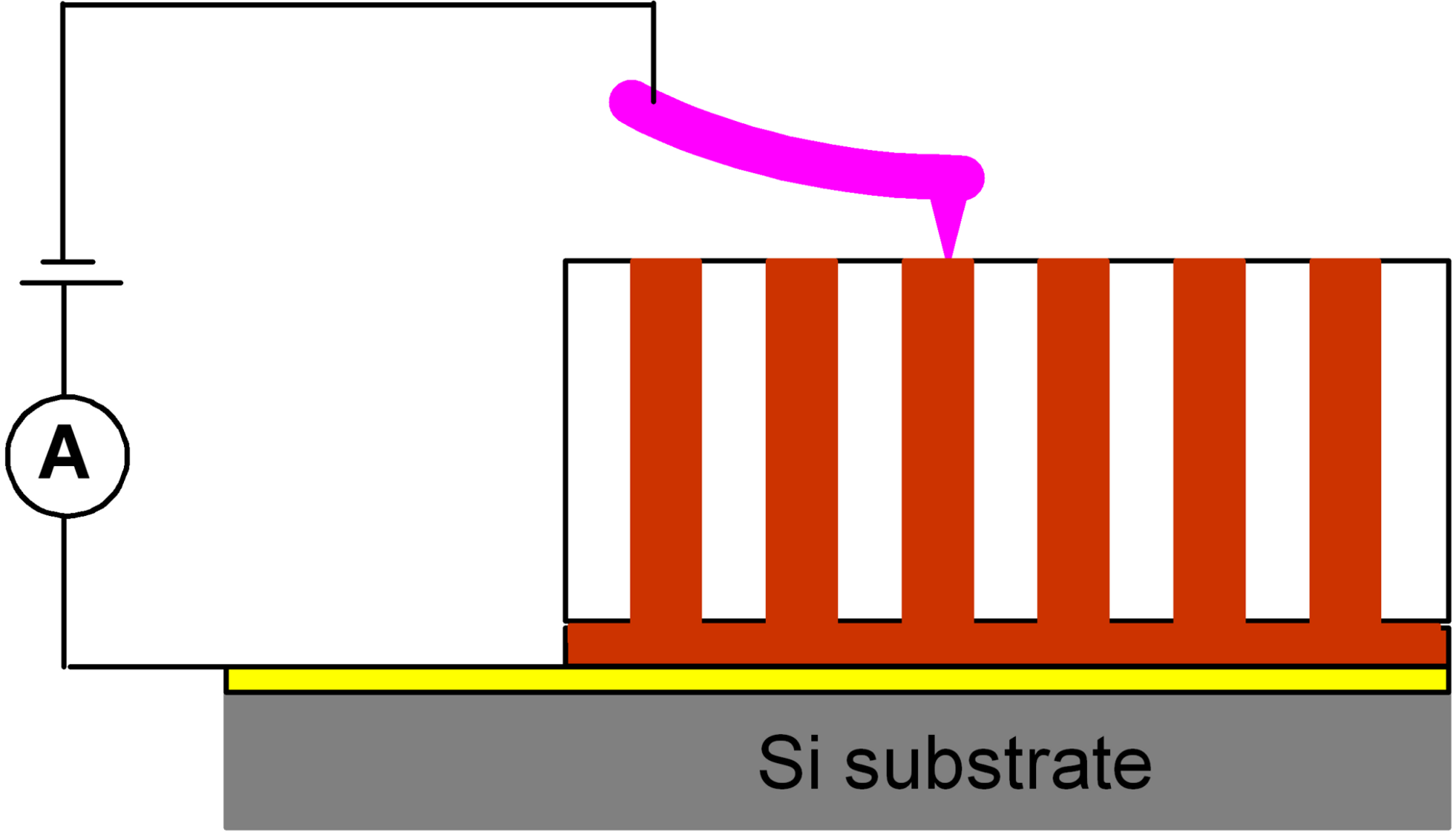


1. Process of fabricating individually addressable nanowire patterns on Si substrate


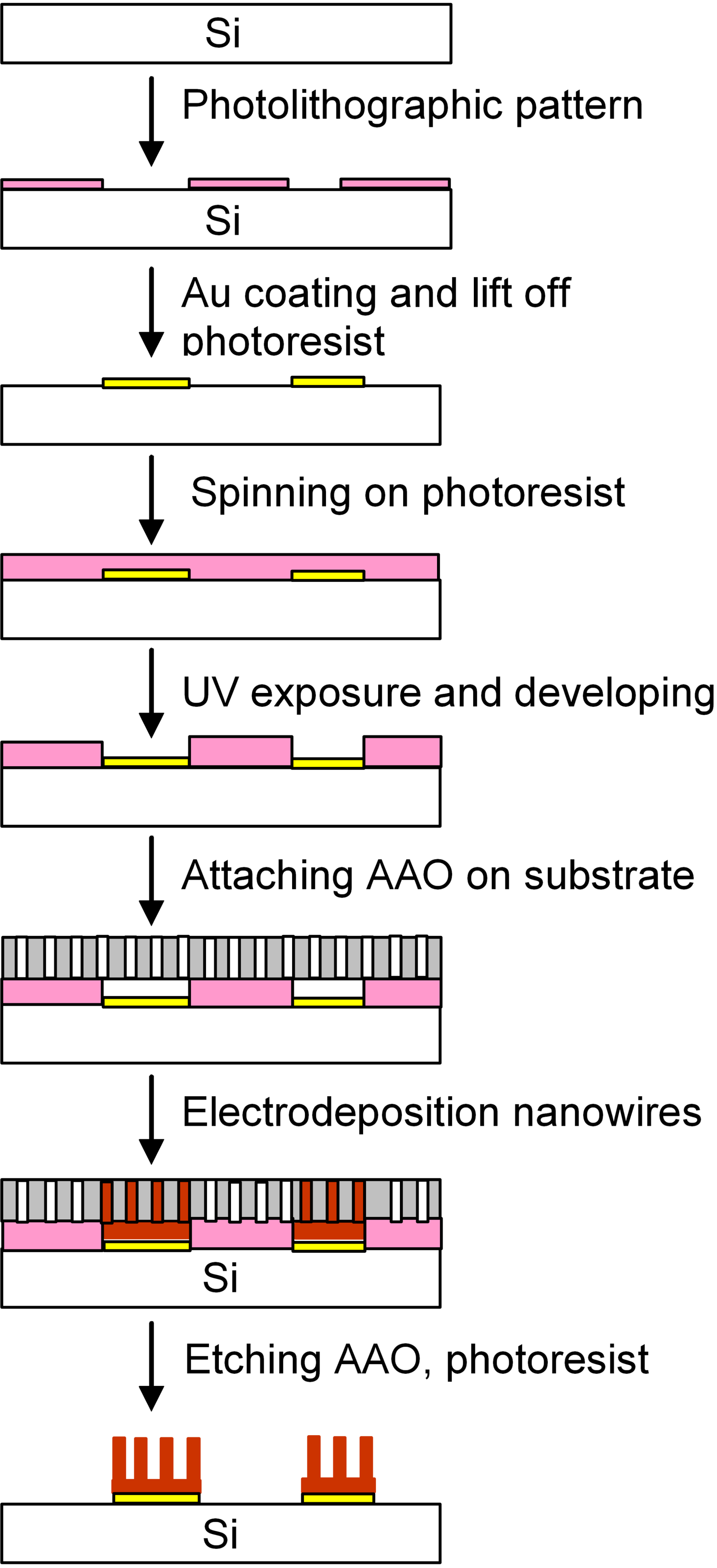

Supplement: Additional file 1 — Electronic Supplementary Material. http://www.nanoscalereslett.com/imedia/2066074704584386/supp1.doc. Figure S1, pull-off adhesion tensile test of silver nanowire arrays within AAM template fabricated on Au-coated Si substrate; Figure S2, conductive atomic force microscopy (C-AFM) characterization; and Figure S3, process of fabricating individually addressable nanowire patterns on Si substrate. [file 1556-276X-6-525-S1.DOC]
